# Identification of Novel Diagnostic Markers for Malignant Pleural Mesothelioma Using a Reverse Translational Approach Based on a Rare Synchronous Tumor

**DOI:** 10.3390/diagnostics12020316

**Published:** 2022-01-27

**Authors:** Tomoaki Naka, Yutaka Hatanaka, Yukiko Tabata, Akira Takasawa, Hideo Akiyama, Yasuhiro Hida, Hiromi Okada, Kanako C. Hatanaka, Tomoko Mitsuhashi, Kei Kushitani, Vishwa Jeet Amatya, Yukio Takeshima, Kouki Inai, Kichizo Kaga, Yoshihiro Matsuno

**Affiliations:** 1 Department of Surgical Pathology, Hokkaido University Hospital, Sapporo 060-8648, Japan; t.naka@pop.med.hokudai.ac.jp (T.N.); yhatanaka@huhp.hokudai.ac.jp (Y.H.); yuki_zipper@hotmail.com (Y.T.); atakasawa@sapmed.ac.jp (A.T.); kanno-kanno@med.hokudai.ac.jp (H.O.); kyanack@huhp.hokudai.ac.jp (K.C.H.); mitsut74@huhp.hokudai.ac.jp (T.M.); 2 Department of Diagnostic Pathology, Faculty of Medicine, Hokkaido University, Sapporo 060-8638, Japan; 3 Research Division of Genome Companion Diagnostics, Hokkaido University Hospital, Sapporo 060-8648, Japan; hideo.akiyama.v8@mail.toray; 4 Department of Cardiovascular and Thoracic Surgery, Graduate School of Medicine, Hokkaido University, Sapporo 060-8638, Japan; yhida@med.hokudai.ac.jp (Y.H.); kaga-hmg@med.hokudai.ac.jp (K.K.); 5 New Projects Development Division, Toray Industries, Inc., Kamakura 248-8555, Japan; 6 Department of Pathology, Graduate School of Biomedical and Health Sciences, Hiroshima University, Hiroshima 734-8551, Japan; kkushi@hiroshima-u.ac.jp (K.K.); amatya@hiroshima-u.ac.jp (V.J.A.); ykotake@hiroshima-u.ac.jp (Y.T.); koinai@byouri.co.jp (K.I.); 7 Pathological Diagnostic Center, Inc., Hiroshima 730-0029, Japan

**Keywords:** malignant pleural mesothelioma, gene expression profiling, immunohistochemistry

## Abstract

Although the routine use of immunohistochemistry has improved the accuracy of histopathologic diagnosis in clinical practice, new methods for discovering novel diagnostic markers are still needed. We sought new diagnostic markers for malignant pleural mesothelioma (MPM) using a reverse translational approach with limited archival tissues from a very rare case. Total RNA extracted from formalin-fixed paraffin-embedded (FFPE) tissues of a synchronous collision tumor consisting of MPM and pulmonary adenocarcinoma (PAC) was employed for gene expression profiling (GEP) analysis. Among the 54 genes selected by GEP analysis, we finally identified the following two candidate MPM marker genes: *PHGDH* and *TRIM29*. Immunohistochemical analysis of 48 MM and 20 PAC cases showed that both PHGDH and TRIM29 had sensitivity and specificity almost equivalent to those of calretinin (sensitivity 50% and 46% vs. 63%, and specificity 95% and 100% vs. 100%, respectively). Importantly, of the 23 epithelioid MMs, all 3 calretinin-negative cases were positive for TRIM29. These two markers may be diagnostically useful for immunohistochemical distinction between MPMs and PACs. This successful reverse translational approach based on FFPE samples from one very rare case encourages the further use of such samples for the development of novel diagnostic markers.

## 1. Introduction

Malignant mesothelioma (MM) is a relatively rare disease, but the number of deaths from this disease has increased over the years [1]. MM has a longer incubation period than other diseases, and the incubation period from the initial exposure to asbestos to the onset of MM is thought to be about 40 years on average [2,3]. Therefore, even in the present era, when the use of asbestos is banned, the number of deaths from MM in the world is estimated to continue to increase, and it is thought that the number of deaths will reach its peak in Japan, for example, from 2030 to 2034 [1]. Because of the limited treatment options, survival time and cost, MM is a major problem throughout the world [4]. Therefore, research leading to the early diagnosis and early treatment of MM is important. The aim of this study is to examine new methods for discovering novel diagnostic markers of MM, and to confirm the usefulness of these novel biomarkers.

Paraffin section immunohistochemistry (IHC) has now become indispensable in the practice of surgical pathology. We routinely rely on IHC to assist us in the diagnosis and classification of neoplasms. In addition to the conventional method of collecting many cases and simply comparing them, we think that a new method is necessary for the discovery of novel diagnostic markers.

In recent years, gene expression profiling (GEP) technology has become a key tool for the discovery of novel biomarkers [5,6,7,8,9,10,11,12,13], and it is readily applicable for analysis based on formalin-fixed paraffin-embedded (FFPE) tissues [14,15]. We have encountered a very rare example of a synchronous collision tumor consisting of both malignant pleural mesothelioma (MPM) and pulmonary adenocarcinoma (PAC) lesions, each of which showed the typical morphology and immunophenotype. In the present study, we employed this unique case to search for diagnostic markers that would allow distinction between MPM and PAC using GEP analysis for both tumors. From the microarray-based expression profile, we found 54 genes that showed higher expression in the MPM lesion than in the PAC lesion. Among them, *PHGDH* and *TRIM29* were selected and validated by qRT-PCR, and the immunohistochemical expression of both PHGDH and TRIM29 was examined using specific antibodies in a series of resected human MM and PAC tissues. In addition, the expression of both MPM marker genes was examined in various histologic types of human tumors and non-tumor tissues. 

## 2. Materials and Methods

### 2.1. A Synchronous Collision Tumor Composed of Malignant Pleural Mesothelioma and Pulmonary Adenocarcinoma

**Figure 1:**
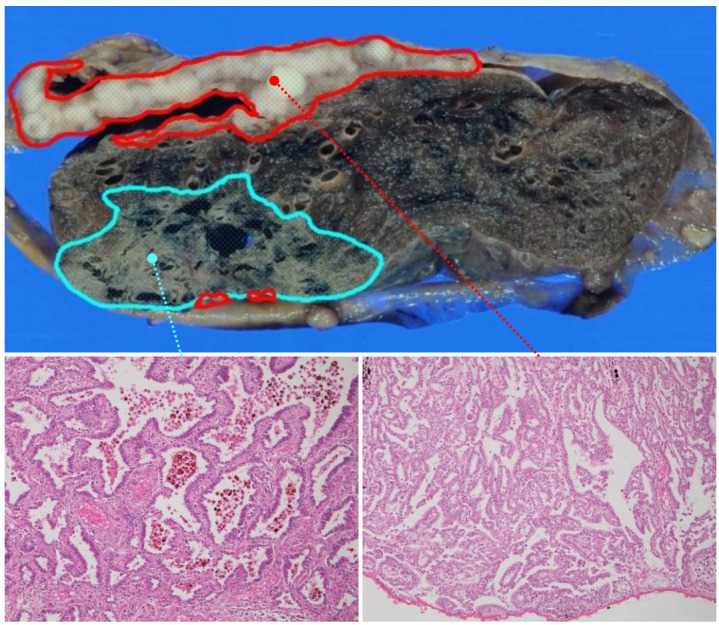
Macroscopy and histology of the synchronous collision tumor consisting of MPM and PAC. Macroscopic and microscopic representation of the tumor. Macroscopically, the lesion appears to consist of the following two components: MPM (encircled by red lines) and PAC (blue lines). Microscopy revealed that these MPM and PAC lesions each exhibited typical histological features.

A collision tumor (epithelioid MPM and PAC) in the right lung of a 77-year-old man, who had a history of long-term smoking and asbestos exposure, was surgically resected at Hokkaido University Hospital. This unusual case has been reported previously and described in detail [16]. Each of the two tumors exhibited a typical immunophenotype and genetic alterations (calretinin^+^/podoplanin^+^/9q21 loss in MPM and TTF-1^+^/Ber-EP4^+^ in PAC, respectively) (Figure 1). FFPE tissue specimens from these tumors were used for further analyses. 

### 2.2. GEP Analysis

Total RNA was extracted from FFPE tissues of each of the two lesions separately using the RNeasy kit (Qiagen, Valencia, CA, USA). RNA quantity was measured using a NanoDrop ND-2000C. RNA integrity number (RIN) was assessed with an Agilent Bioanalyzer 2100. GEP analysis was performed using the 3D-Gene human Oligo chip 25k (Toray Industries, Inc, Tokyo, Japan) with RNA extracts. Briefly, antisense RNA (ds-aRNA) was synthesized from total RNA, amplified, and Cy5-labeled in accordance with the manufacturer’s protocol. Microarrays were then hybridized and the slides were scanned using Scan Array Lite (Perkin Elmer, Waltham, MA, USA). Expression data were subjected to global normalization, and differentially expressed genes were identified through fold change filtering.

### 2.3. qRT-PCR Analysis

The MPM cell lines MESO-1 and MESO-4, and the PAC cell lines A549 and H1299 were used for validation by qRT-PCR analysis. MESO-1 and MESO-4 were grown in RPMI-1640 (Sigma-Aldrich Japan, Tokyo, Japan), and A549 and H1299 were cultured in D-MEM (Gibco BRL, Grand Island, NY, USA), both of which were supplemented with 10% fetal bovine serum (FBS) and 1% penicillin/streptomycin. All cell lines were maintained in a humidified incubator with 5% CO_2_ in air at 37 °C.

Total RNAs from the cell lines and FFPE tissues were extracted using Trizol reagent (Invitrogen Life Technologies, Carlsbad, CA, USA) and a ReliaPrep FFPE total RNA Miniprep System (Promega, Fitchburg, WI, USA), respectively. RNA quantity and integrity were evaluated as described above. cDNA was synthesized with a Transcript First Strand cDNA Synthesis Kit (Roche, Indianapolis, IN, USA) in accordance with the manufacturer’s protocol. The gene expression levels of *PHGDH* and *TRIM29* were analyzed by quantitative reverse transcription–polymerase chain reaction (qRT–PCR) using QuantiTect SYBR Green PCR Mix (Qiagen) and the following primers: PHGDH (forward) 5′- CACAGGTGTGGACAATGTGGAT-3′, (reverse) 5′- GCCAGGCACATGATCATTCC-3′; TRIM29 (forward) 5′- AAGGTGCTGCATGAGGACAAG-3′, (reverse) 50- TGCACCAAATTCCTGCAGAA-30; GAPDH as a control (forward) 5′-ATCAAGTGGGGCGATGCTG-30, (reverse) 5′-ACCCATGACGAACATGGGG-3′. Cycling conditions were those recommended by the manufacturer for Step One Plus (Applied Biosystems, Foster City, CA, USA). The PCR conditions were as follows: initial denaturation at 95 °C for 15 min, followed by 40 cycles of denaturation at 94 °C for 30 s, annealing at 60 °C for 30 s, and extension at 72 °C for 30 s.

### 2.4. Immunohistochemical Evaluation of MM and PAC Cases

A total of 48 cases of MM (23 epithelioid, 13 biphasic and 12 sarcomatoid) comprising 45 pleural cases, and one case each from the pericardium, peritoneum and tunica vaginalis, and 20 cases of PAC obtained between January 1994 and December 2010 from Hokkaido University Hospital and Hiroshima University Hospital were used in this study (Table 1). The mean age of all MM patients was 63 (39–83). The mean age of the patients with epithelioid, biphasic, and sarcomatoid MMs was 60 (39–80), 62 (52–73), and 69 (66–83), respectively. Forty-four cases were male and all four females were epithelioid. The mean age of PAC patients was 66 (53–78), with 14 males. The FFPE tissue sections were deparaffinized in xylene and rehydrated through a graded ethanol series. Heat-induced antigen retrieval was carried out in high-pH antigen retrieval buffer (Dako, Glostrup, Denmark). Endogenous peroxidase was blocked by incubation in 3% H_2_O_2_ for 5 min. Details of the primary antibodies used are listed in Table 2. These sections were visualized by the HRP-labeled polymer method (Dako EnVision FLEX system) and an automated immunostaining system (Dako Autostainer Link). Immunostained sections were counterstained with hematoxylin, dehydrated in ethanol and cleared in xylene.

In this study, we evaluated immunohistochemical staining in terms of both intensity and the proportion of positive tumor cells. The intensity of staining was evaluated using a sliding scale ranging from 0 to 3+ (0 = negative staining, 1+ = weak, 2+ = intermediate, and 3+ = strong). For PHGDH and calretinin, intensity scores of 0 and 1+ were categorized as negative. When 2+ or 3+ cytoplasmic staining was observed in more than 10% of tumor cells, the case was judged as positive. For TRIM29, an intensity score of 0 was categorized as negative. When 1+, 2+ or 3+ cytoplasmic staining was observed in more than 10% of tumor cells, the case was judged as positive. For calretinin, only cases showing unequivocal staining in both the nucleus and cytoplasm were deemed positive. Two pathologists (TN and YM), who were blinded to the patients’ clinical information, examined the cases independently and scored them. Any differences in their interpretation were resolved by consensus after a joint review.

### 2.5. Immunohistochemical Analysis of Various Tissues and Organs

Tumors and adjacent non-neoplastic tissues were retrieved from surgical specimens in the pathology files of Hokkaido University Hospital covering the period from 2000 to 2003. The tissue specimens had been fixed in 10% neutral buffered formalin for 24 to 48 h, and then embedded in paraffin wax. A tissue microarray (TMA) was prepared using the following procedure. Specifically, after evaluation of a hematoxylin and eosin-stained section from each FFPE tissue block to locate representative areas for further analysis, needle core samples (1.0 mm) were cut out from the corresponding areas of the block, and then placed at prespecified coordinates in recipient paraffin array blocks using a manual tissue microarrayer (Sakura Finetek Japan, Tokyo). Thus, array blocks each containing between 119 and 144 cores were constructed, covering a total of 266 FFPE tumor tissue samples derived from 16 types of primary neoplasm and their non-neoplastic counterpart. Tumor types and cases examined included squamous cell carcinoma (SCC) of the lung (9 cases), urothelial carcinoma (10 cases), prostatic adenocarcinoma (10 cases), clear cell renal cell carcinoma (10 cases), hepatocellular carcinoma (10 cases), gastric adenocarcinoma (10 cases—6 diffuse type, 3 intestinal type, and 1 gastric carcinoma with lymphoid stroma), colon adenocarcinoma (10 cases), pancreatic duct carcinoma (10 cases), invasive ductal carcinoma of the breast (17 cases—10 hormone receptor- and/or HER2-positive, and 7 triple-negative), uterine cervical squamous cell carcinoma and adenocarcinoma (7 and 2 cases, respectively), endometrial adenocarcinoma (10 cases—7 endometrioid, 2 serous, and 1 clear), ovarian adenocarcinoma (10 cases— [11] 4 serous, 4 clear, 1 endometrioid, and 1 mucinous), gastrointestinal stromal tumor (GIST, 5 cases), gastrointestinal neuroendocrine tumor (NET, 5 cases), and malignant lymphoma (9 cases—3 follicular, 2 marginal zone, and 4 diffuse large B-cell). Immunohistochemical staining of PHGDH and TRIM29 using TMA sections and its evaluation were performed as described above.

## 3. Results

### 3.1. Differential GEP Analysis of MPM and PAC

**Figure 2:**
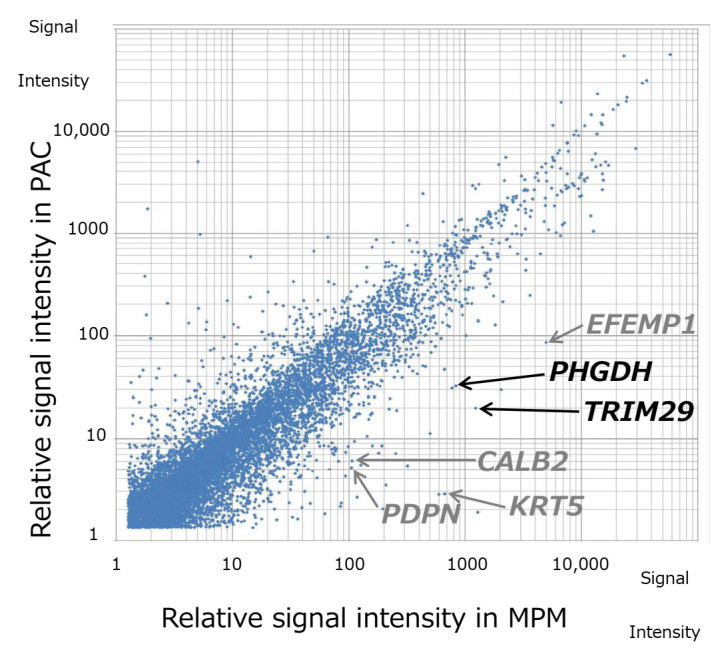
Gene expression profiling of the synchronous collision tumor consisting of MPM and PAC. Whole-genome RNA expression profiling was performed on the MPM and PAC lesions in samples from the tumor. A total of 949 RNAs were identified through microarray analysis, and 54 genes showed more than 8-fold upregulation in the MPM lesion relative to the PAC lesion. Gene expression profiling identified two novel MPM marker genes, *PHGDH* and *TRIM29*. The genes identified by gene expression profiling also included well-known MPM markers such as *CALB2* (calretinin), *PDPN* (podoplanin), *KRT5* (CK5) and *EFEMP1* (fibulin-3).

The RIN values for total RNA extracted from the MPM and PAC lesions in the collision tumor were 6.2 and 2.8, respectively. Although lower RNA integrity was observed in the PAC than in the MPM, GEP analysis was successful for both lesions. A total of 949 RNAs were identified through the analysis (Figure 2 and Supplementary Table S1). Of these, the expression levels of 718 genes were increased and 231 were decreased in the MPM lesion, relative to the PAC. Fifty-four genes showing an increment of more than eight-fold in the MPM lesion were extracted.

### 3.2. Validation of MPM Markers

**Figure 3:**
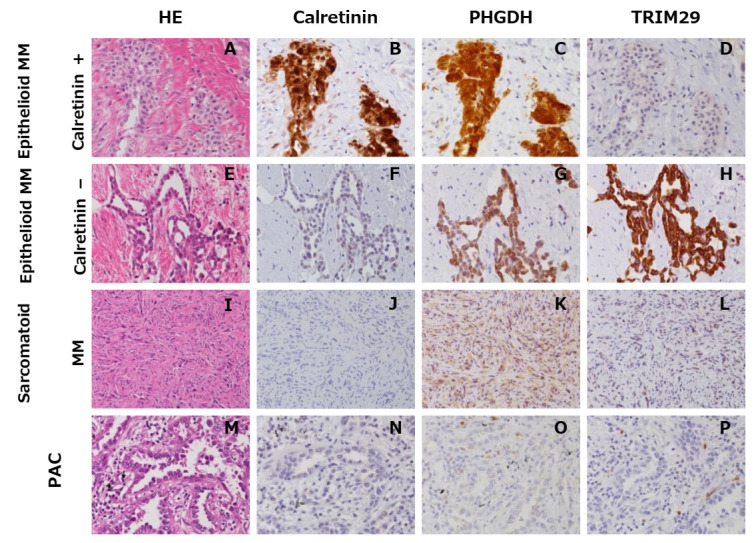
Immunohistochemical staining panel for PHGDH, TRIM29 and calretinin in representative cases of MM and PAC. Representative immunohistochemical staining patterns of PHGDH and TRIM29 in the following different cases of MMs and PACs: calretinin-positive epithelioid MM (**A**–**D**), calretinin-negative epithelioid MM (**E**–**H**), sarcomatoid MM (**I**–**L**), and PAC (**M**–**P**). PHGDH and TRIM29 show strong cytoplasmic staining (**C**,**G**,**H**). In the vast majority of epithelioid MMs, positive immunoreactivity for both PHGDH and TRIM29 was observed. All 3 calretinin-negative epithelioid MM cases were TRIM29-positive (F and H). Sarcomatoid MMs and PAC were rarely stained for PHGDH, TRIM29, or calretinin (**J**–**L**,**N**–**P**).

From the extracted genes, we excluded those that had previously been reported to be MPM markers, including *KRT5*, *CALB2*, *PDPN, EFEMP1, CD44* (encoding cytokeratin 5 (CK5), calretinin, podoplanin, fibulin-3 [17,18], and CD44, respectively) and genes whose function remained uncharacterized upon completion of the GEP analysis. Subsequently, nine genes (*TRIM29, PHGDH, SIGLEC11, RBP4, RGMA, PRSS11, KLK11, SLC9A3R1*, and *ID2*) selected from those remaining were validated (data not shown). Based on the results of qRT-PCR and the immunohistochemical analyses of the cell lines and the FFPE tissues from the synchronous collision tumor, we finally identified two novel MPM marker genes, *PHGDH* and *TRIM29* (Figure 3 and Supplementary Figure S1).

### 3.3. Immunohistochemical Evaluation of PHGDH and TRIM29

To evaluate the immunohistochemical utility of PHGDH and TRIM29 for differential diagnosis between MM and PAC, we compared these markers with calretinin next, which is one of the most widely used markers of mesothelial cells. Figure 3 shows the representative immunohistochemical staining patterns for PHGDH and TRIM29 in MM and PAC tissues. PHGDH and TRIM29 showed a cytoplasmic staining pattern (Figure 3C,G,H). In epithelioid MMs, positive immunoreactivity was observed in 74% of cases for both PHGDH and TRIM29 (Table 3). Sarcomatoid MMs were rarely stained for PHGDH, TRIM29 and calretinin (0%, 0%, and 8%, respectively). In biphasic MMs, positivity for PHGDH and TRIM29 was substantially restricted to the epithelioid areas of those tumors. Only 1 out of 20 PAC cases showed intermediate (2+) PHGDH immunostaining in a limited area, showing a micropapillary growth pattern. As immunohistochemical markers for MM, PHGDH and TRIM29 were quite comparable to calretinin in terms of sensitivity (50% and 46% vs. 63%, respectively) and equivalent in terms of specificity (95% and 100% vs. 100%, respectively). It was particularly noteworthy that all 3 calretinin-negative cases among 23 epithelioid MMs were positive for TRIM29 (Figure 3F,H). Peritoneal MM was positive for TRIM29 and negative for PHGDH, whereas tunica vaginalis MM yielded an opposite result (negative for TRIM29 and positive for PHGDH). Pericardial MM was negative for both PHGDH and TRIM29.

### 3.4. Expression of PHGDH and TRIM29 in Tumors other than MM and in Normal Tissues

**Figure 4:**
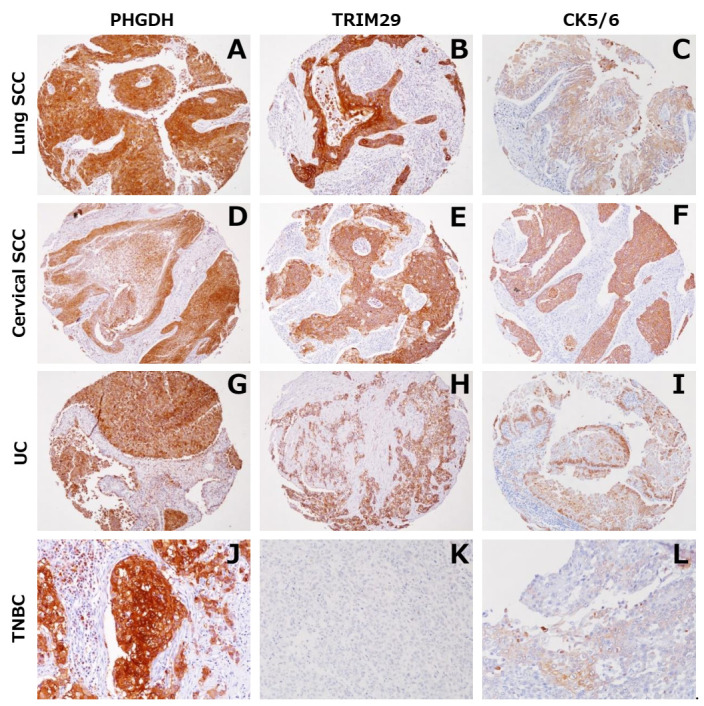
Immunohistochemical staining for PHGDH and TRIM29 in tumors other than MM in TMA. Representative immunohistochemical staining patterns in tumor samples for PHGDH (**A**,**D**,**G**,**J**), TRIM29 (**B**,**E**,**H**,**K**) and CK5/6 (**C**,**F**,**I**,**L**). PHGDH, TRIM29 and CK5/6 were occasionally detected in squamous cell carcinoma of the lung (**A**–**C**), squamous cell carcinoma of the uterine cervix (**D**–**F**) and urothelial carcinoma (**G**–**I**). PHGDH and CK5/6 were expressed significantly in triple-negative invasive ductal carcinoma, while TRIM29 was not (TNBC; **J**–**L**).

To investigate the expression of PHGDH and TRIM29 in various types of tumors other than MM and in normal tissues, we used a TMA consisting of 16 types of primary tumor, as described in the Materials and Methods section. As shown in Figure 4 and Table 4, both PHGDH and TRIM29 exhibited significant expression in SCC of the lung and urothelial carcinoma. In addition, PHGDH was expressed in prostatic adenocarcinoma, invasive ductal carcinoma of the breast, endometrial adenocarcinoma and ovarian adenocarcinoma. TRIM29 was expressed in SCC of the uterine cervix. Among seven cases of triple-negative invasive ductal carcinoma of the breast (TNBC), five and two cases showed positive staining for PHGDH and TRIM29, respectively.

PHGDH and TRIM29 were occasionally detected in the normal squamous epithelium of the uterine cervix, prostate basal cells, bronchial basal cells and mesothelial cells, which are also positive for CK5/6 (Supplementary Figure S2).

## 4. Discussion

In the present study, we demonstrated that a reverse translational approach has great potential for the discovery of novel diagnostic markers, by focusing on a rare single case of collision tumor comprising two tumors that had arisen concurrently from two different types of cell. Unlike the general comparative GEP approach, the present procedure allows minimization of the cohort/sample size for analysis, thus avoiding any bias due to case selection. The RIN values of the two samples used here were 6.2 for MPM and 2.8 for PAC. Since the PAC lesion was overlooked upon initial gross examination and tissue sampling was delayed, the lower RNA quality of the PAC lesion, relative to the MPM, would have been attributable to prolonged formalin fixation. Despite this, our GEP analysis was successful, not only in terms of searching for novel MPM markers, but also in detecting already established markers, such as cytokeratin 5, calretinin, podoplanin, fibulin-3 [17,18] and CD44, as described above. These results indicate that the present approach, which used routine FFPE tissues from a very rare case, even with limited RNA quality, would be able to provide considerably reliable data for the exploration of novel biomarkers.

In this study, we performed GEP analysis using epithelioid MPM and found novel diagnostic markers, PHGDH and TRIM29, that were positive for epithelioid and biphasic MMs. Unfortunately, PHGDH and TRIM29 were negative for sarcomatoid MM, but we believe that analysis of sarcomatoid MM using the same technique should also find new diagnostic markers for sarcomatoid MM. Since PHGDH and TRIM29 are expressed in the epithelioid MM and not in the sarcomatoid MM, they may play an important role in the development and proliferation of the epithelioid MM. The details have not been examined this time and need further study.

No previous report has indicated that MPM expresses PHGDH or TRIM29 [12,13,18]. Nevertheless, both PHGDH and TRIM29 showed better immunohistochemical specificity and sensitivity than expected, despite the use of antibodies that are not really suited for routine FFPE immunohistochemistry. For example, the sensitivity and specificity of TTF-1 for the diagnosis of PACs differs depending on the antibody clone employed [19]. It is noteworthy that TRIM29 was detected in all cases of calretinin-negative MM, for which diagnosis has been challenging up to now. The development of antibodies suitable for routine testing would improve diagnostic specificity and sensitivity, and TRIM29 may become a diagnostic marker for calretinin-negative MPM, which is currently difficult to diagnose.

Phosphoglycerate dehydrogenase (PHGDH), encoded by the *PHGDH* gene located on chromosome 1p12, controls flux from the glycolytic pathway into the serine biosynthesis pathway [20,21]. RNA for *PHGDH* was found at high levels in the prostate, testis, ovary, brain, liver, kidney, and pancreas. Lower levels were also found in the mucosal epithelial lining of the colon, and weaker expression was also evident in the thymus, small intestine, and heart [22]. It is reported that *PHGDH* is overexpressed in a subset of breast cancer, cervical cancer, and melanoma [23]. On the other hand, TRIM29 (also known as ataxia-telangiectasia group D complementing, or ATDC) is a member of the tripartite motif (TRIM) protein family, encoded by the *TRIM29* gene located on chromosome 11q23. It contains multiple zinc-finger motifs and a leucine zipper motif, and, thus, may act as a transcriptional regulatory factor [24,25]. *TRIM29* is normally expressed in the placenta, lung, thymus, prostate, testis, and colon, whereas it is undetectable in the heart, brain, skeletal muscle, pancreas, spleen, ovary, and small intestine [26].

Interestingly, previous studies have shown that both PHGDH and TRIM29 are characteristically expressed in a subset of epithelial cells, such as the normal squamous epithelium, considered to be positive for CK5 [20,24,27]. Proteomic profiling in breast cancers has identified PHGDH as one of the proteins expressed specifically in triple-negative breast cancers, which often have a basal-like phenotype defined by positivity for EGFR and CK5 [20,27]. PHGDH is also immunohistochemically expressed in normal and neoplastic uterine cervical cells [23]. Moreover, *TRIM29* is specifically expressed in prostate basal cells, uterine cervical cancer cells and basal subtype bladder cancer cells [28,29,30]. TRIM29 appears to be a highly sensitive marker for cells showing squamous differentiation in urinary bladder carcinoma [24]. In the present study, expression of PHGDH and TRIM29 was observed in CK5/6-positive squamous epithelium of the uterine cervix, prostate basal cells, bronchial basal cells and mesothelial cells, in addition to MM cells. PHGDH was expressed in triple-negative breast cancers and squamous cell carcinomas, and TRIM29 was expressed in squamous cell carcinomas of the uterine cervix and urothelial carcinomas, which are findings that are highly compatible with the previous studies mentioned above. The fact that PHGDH and TRIM29 are often co-expressed with CK5/6 in a range of cell types implies that there may be some linkage between the expressions of these three molecules.

Recently, it has been reported that PHGDH and TRIM29 are expressed in lung adenocarcinoma, and are indicators of poor prognosis [31,32,33,34]. As the present study was focused on the development of novel diagnostic markers that could be used for immunohistochemistry, we did not investigate their possible prognostic application. Therefore, the method used for the evaluation of immunohistochemical staining differed from those in previous studies. That is, Song X et al. judged nuclear staining to indicate positivity for TRIM29, whereas in the present study, we evaluated cytoplasmic staining [31]. The clone of PHGDH used in the present study also differed from that used by Zhang et al. or Dong et al. [27,29]. Although Zhu et al. appeared to use an anti-PHGDH antibody from Abcam, they did not state the clone chosen [33]. As Abcam has released several PHGDH clones, different results might have been obtained if different clones had been used. Further research on the expression of PHGDH and TRIM29 in PAC is therefore warranted.

Although this study revealed that PHGDH and TRIM29 may be diagnostically useful for immunohistochemical distinction between MPMs and PACs, it has several limitations. First, we retrospectively collected the cohort data, and selection bias was not completely eliminated. Second, since the expression of PHGDH and TRIM29 in tumors other than MM and in normal tissues were examined by TMA, intratumoral heterogeneity was not examined.

In summary, we have reported, for the first time, the possible utility of PHGDH and TRIM29 as diagnostic markers for the distinction between MPMs and PACs. Our reverse translational approach, based on a very rare single case of collision tumor, encourages the further use of such samples for the development of novel diagnostic markers.

## Figures and Tables

**Table 1:** Clinical characteristics in malignant mesotheliomas (MMs) and PACs.

	MM				PAC (*n* = 20)
	Total (*n* = 48)	Epithelioid (*n* = 23)	Biphasic (*n* = 13)	Sarcomatoid (*n* = 12)	
Mean age (range)	63 (39–83)	60 (39–80)	62 (52–73)	69 (66–83)	66 (53–78)
Male/Female	44/4	19/4	13/0	12/0	14/6

**Table 2:** Antibodies employed for immunohistochemistry (IHC).

Antibody	Source	Clone	Dilution	Antigen Retrieval
PHGDH	abcam	ab57030	1:100	Dako TRS High pH
TRIM29	abcam	ab22207	1:50	Dako TRS High pH
Calretinin	Dako	DAK-Calret 1	1:50	Leica BOND ER2

**Table 3:** Immunohistochemical expression in MMs and PACs.

	PHGDH Positive Case/*n* (%)	TRIM29 Positive Case/*n* (%)	Calretinin Positive Case/*n* (%)
MM	25/48 (52)	22/48 (46)	30/48 (63)
Epithelioid	17/23 (74)	17 */23 (74)	20/23 (87)
Biphasic	8/13 (62)	6/13 (46)	9/13 (69)
Sarcomatoid	0/12 (0)	0/12 (0)	1/12 (8)
PAC	1 ^a^/20 (5)	0/20 (0)	0/14 (0)

^a^ One of twenty PAC cases showed intermediate (2+) PHGDH staining, especially tumor cells of the micropapillary subtype. * includes all 3 cases of calretinin-negative MM.

**Table 4:** Immunohistochemical expression in various tumors (TMA).

	PHGDH Positive Case/*n* (%)	TRIM29 Positive Case/*n* (%)
Lung squamous cell carcinoma	6/9 (67)	9/9 (100)
Urothelial carcinoma	7/10 (70)	9/10 (90)
Prostatic adenocarcinoma	9/10 (90)	0/10 (0)
Clear cell renal cell carcinoma	0/10 (0)	0/10 (0)
Hepatocellular carcinoma	2/10 (20)	3/10 (30)
Gastric adenocarcinoma	2/10 (20)	3/10 (30)
Colon adenocarcinoma	0/10 (0)	0/10 (0)
Pancreatic duct carcinoma	2/10 (20)	3/10 (30)
Invasive ductal carcinoma of the breast		
HR- and/or HER2-positive	5/10 (50)	1/10 (10)
Triple-negative	5/7 (71)	2/7 (29)
Uterine cervical squamous cell carcinoma	4/7 (57)	7/7 (100)
Uterine cervical adenocarcinoma	0/2 (0)	1/2 (50)
Endometrial adenocarcinoma	8/10 (80)	2/10 (20)
Ovarian adenocarcinoma	6/10 (60)	1/10 (10)
GIST	0/5 (0)	0/5 (0)
Gastrointestinal NET	0/5 (0)	0/5 (0)
Malignant lymphoma	1/9 (11)	0/9 (0)

RCC, renal cell carcinoma; HR, hormone receptor; GIST, gastrointestinal stromal tumor; NET, neuroendocrine tumor.

## Data Availability

The datasets of the current study are available from the corresponding author on reasonable request.

## Supplementary Materials

The following are available online at https://www.mdpi.com/article/10.3390/diagnostics12020316/s1: Table S1: The RNA expression levels of genes of the synchronous collision tumor consisting of MPM and PAC; Figure S1: validation assay of PHGDH and TRIM29 using qRT-PCR with extracted RNA and immunohistochemistry; Figure S2: immunohistochemical staining for PHGDH and TRIM29 in non-tumor tissues.
