# Supplementary material for: Identification of Novel Diagnostic Markers for Malignant Pleural Mesothelioma Using a Reverse Translational Approach Based on a Rare Synchronous Tumor
Source: Diagnostics (Basel). 2022 Jan 27;12(2):316. doi: 10.3390/diagnostics12020316 (PMC8871196; doi:10.3390/diagnostics12020316)
Supplement: Supplementary file 1 [file diagnostics-12-00316-s001.zip › Supplementary figure (tomoaki naka).pdf]

Supplementary Information

Identification of novel diagnostic markers for malignant pleural mesothelioma using a reverse translational approach based on a rare synchronous tumor

Tomoaki Naka[1,3], Yutaka Hatanaka[1,2], Yukiko Tabata[1,4], Akira Takasawa[1], Hideo Akiyama[2,5], Yasuhiro Hida[4], Hiromi Okada[1], Kanako C. Hatanaka[1,2], Tomoko Mitsuhashi[1], Kei Kushitani[6], Vishwa Jeet Amatya[6], Yukio Takeshima[6], Kouki Inai[6,7], Kichizo Kaga[4] & Yoshihiro Matsuno[1,2,3]§

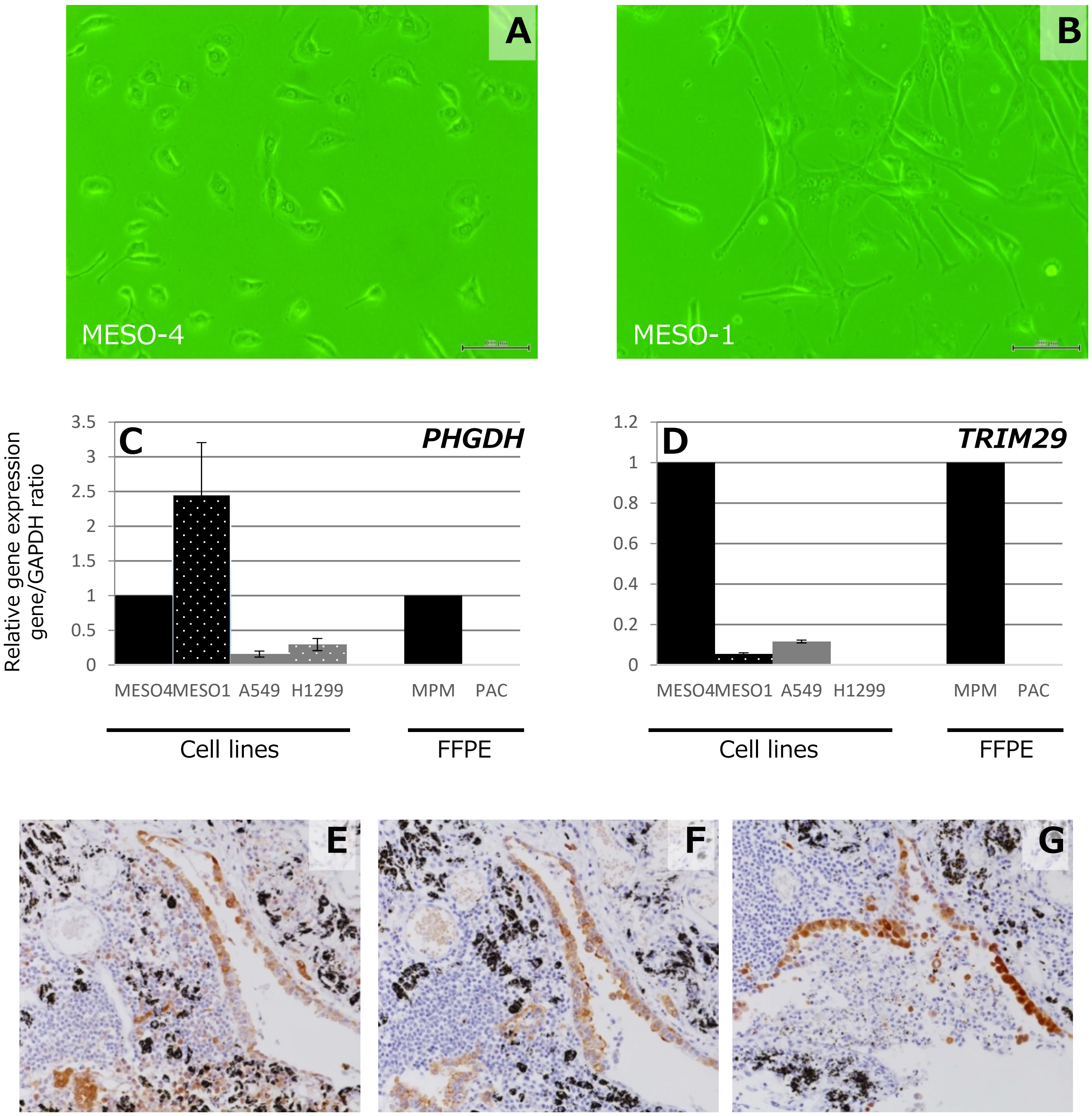

Supplementary Figure S1 Validation assay of *PHGDH* and *TRIM29* using qRT-PCR with extracted RNA and immunohistochemistry

Alterations in the expression of mRNAs for *PHGDH* (C) and *TRIM29* (D) were validated by qRT-PCR in cell lines and FFPE tissues from the synchronous collision tumor. Expression of *PHGDH* and *TRIM29* was markedly enhanced in the MPM cell lines relative to the PAC cell lines: TRIM29 showed much higher expression in MESO-4, with an epithelial-like morphology (A), whereas PHGDH showed much higher expression in MESO-1, with a fibroblast-like morphology (B). We also found significant upregulation of *PHGDH* and *TRIM29* in MPMs relative to PACs, using RNAs extracted from FFPE tissues of the synchronous collision tumor, being consistent with the microarray data. Immunohistochemistry also revealed positivity for PHGDH (E) and TRIM29 (F) in MPM cells, which were also stained for calretinin (G).

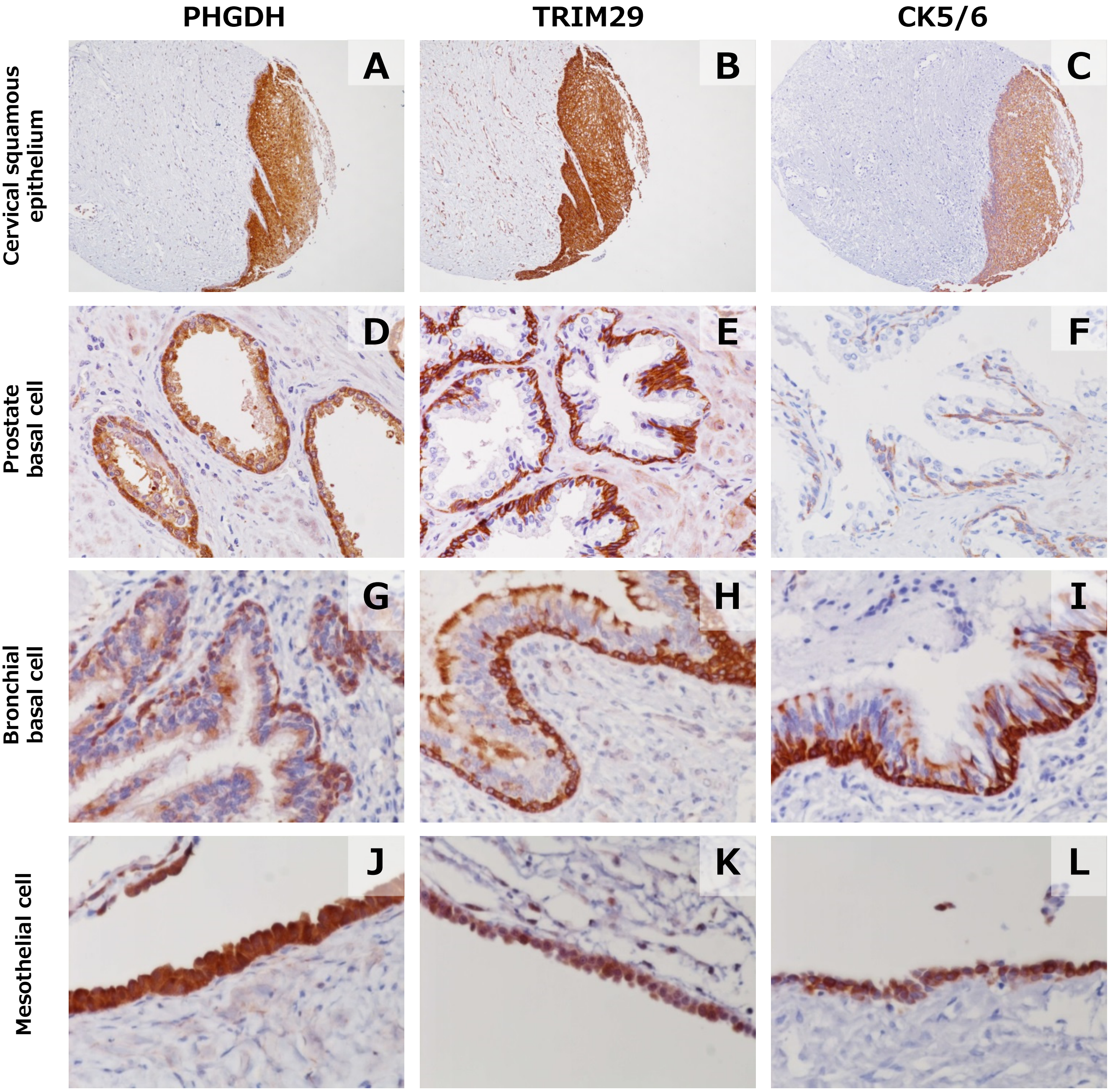

Supplementary Figure S2 Immunohistochemical staining for PHGDH and TRIM29 in non-tumor tissues

Representative immunohistochemical staining patterns in non-tumor samples for PHGDH (A, D, G, J), TRIM29 (B, E, H, K) and CK5/6 (C, F, I, L). PHGDH, TRIM29 and CK5/6 were occasionally detected in the normal squamous epithelium of the uterine cervix (A-C), prostate basal cells (D-F), bronchial basal cells (G-I) and mesothelial cells (J-L).
